# Bacteria-engineered porous sponge for hemostasis and vascularization

**DOI:** 10.1186/s12951-022-01254-7

**Published:** 2022-01-21

**Authors:** Jie Bian, Luhan Bao, Xiaokang Gao, Xiao Wen, Qiang Zhang, Jinhua Huang, Zhenghui Xiong, Feng F. Hong, Zili Ge, Wenguo Cui

**Affiliations:** 1 Department of Stomatology, The First Affiliated Hospital of Soochow University, 899 Pinghai Road, Suzhou, 215006 Jiangsu People’s Republic of China; 2 Department of Stomatology, The Affiliated Hospital of Yangzhou University, Yangzhou University, 368 Hanjiang Middle Road, Yangzhou, 225000 Jiangsu People’s Republic of China; 3 Department of Orthopaedics, Shanghai Key Laboratory for Prevention and Treatment of Bone and Joint Diseases, Shanghai Institute of Traumatology and Orthopaedics, Ruijin Hospital, Shanghai Jiao Tong University School of Medicine, 197 Ruijin 2nd Road, Shanghai, 200025 People’s Republic of China; 4 Department of General Surgery, The Affiliated Hospital of Yangzhou University, Yangzhou University, 368 Hanjiang Middle Road, Yangzhou, 225000 Jiangsu People’s Republic of China; 5 Scientific Research Base of Bacterial Nanofiber Manufacturing and Composite Technology, College of Chemistry, Chemical Engineering and Biotechnology, Donghua University, North Ren Min Road 2999, Shanghai, 201620 People’s Republic of China

**Keywords:** Sponge, Hemostasis, Vascularization, Oxidized bacterial nanocellulose, Deferoxamine

## Abstract

**Background:**

Hemostasis and repair are two essential processes in wound healing, yet early hemostasis and following vascularization are challenging to address in an integrated manner.

**Results:**

In this study, we constructed a hemostatic sponge OBNC-DFO by fermentation of *Komagataeibacter* *xylinus* combined with TEMPO oxidation to obtain oxidized bacterial nanocellulose (OBNC). Then angiogenetic drug desferrioxamine (DFO) was grafted through an amide bond, and it promoted clot formation and activated coagulation reaction by rapid blood absorption due to the high total pore area (approximately 42.429 m^2^/g measured by BET). The further release of DFO stimulated the secretion of HIF-1α and the reconstruction of blood flow, thus achieving rapid hemostasis and vascularization in damaged tissue. This new hemostatic sponge can absorb water at a rate of approximate 1.70 g/s, rapidly enhancing clot formation in the early stage of hemostasis. In vitro and in vivo coagulation experiments (in rat tail amputation model and liver trauma model) demonstrated superior pro-coagulation effects of OBNC and OBNC-DFO to clinically used collagen hemostatic sponges (COL). They promoted aggregation and activation of red blood cells and platelets with shorter whole blood clotting time, more robust activation of endogenous coagulation pathways and less blood loss. In vitro cellular assays showed that OBNC-DFO prevailed over OBNC by promoting the proliferation of human umbilical vein endothelial cells (HUVECs). In addition, the release of DFO enhanced the secretion of HIF-1α, further strengthening vascularization in damaged skin. In the rat skin injury model, 28 days after being treated with OBNC-DFO, skin appendages (e.g., hair follicles) became more intact, indicating the achievement of structural and functional regeneration of the skin.

**Conclusion:**

This hemostatic and vascularization-promoting oxidized bacterial nanocellulose hemostatic sponge, which rapidly activates coagulation pathways and enables skin regeneration, is a highly promising hemostatic and pro-regenerative repair biomaterial.

**Graphical Abstract:**

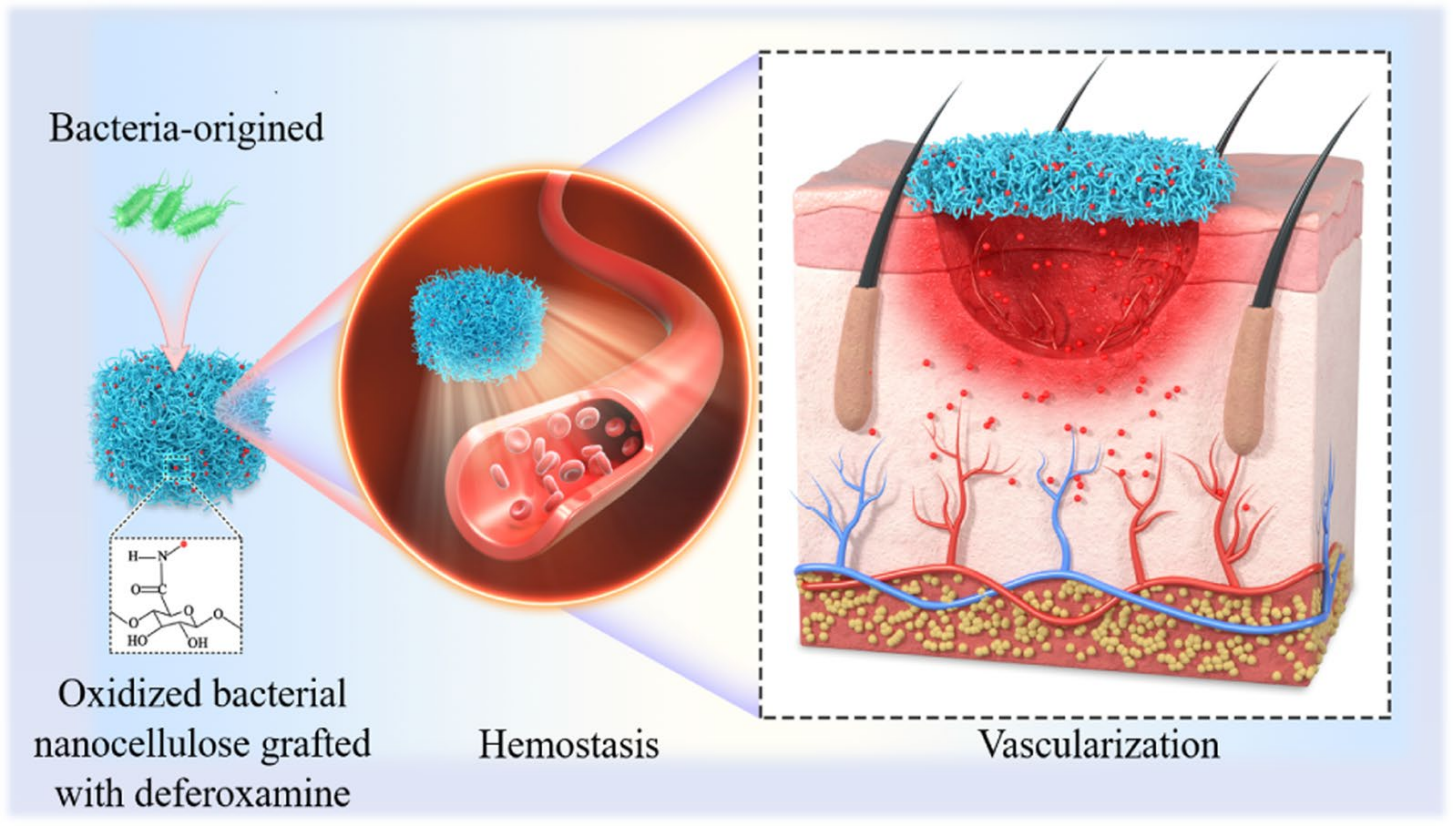

**Supplementary Information:**

The online version contains supplementary material available at 10.1186/s12951-022-01254-7.

## Background

Wound healing, in a broad sense, includes two fundamental processes: hemostasis and repair [1]. Post-traumatic hemorrhage is the primary cause of morbidity and mortality in civilians and military populations, accounting for approximately 30% of all trauma deaths worldwide; therefore, early and rapid hemostasis plays a decisive role in the survival and optimal recovery of patients with significant trauma [2–5]. The hemostasis mechanism in the human body is a complex, spatiotemporally regulated response involving various blood cells (platelets, red blood cells, white blood cells, etc.) and plasma components (fibrinogen, coagulation factors, etc.) [2, 6]. Traditional methods of halting bleeding, such as local compression bandaging, dressings filling and tourniquets applying [7], may cause distal tissue ischemia and metabolic abnormalities, leading to severe complications. Intravenous administration of fibrinogen and coagulation factors can significantly modulate the coagulation cascade process, having noticeable effects in controlling bleeding in trauma and injuries. Such products have been extensively studied in preclinical animal models as well as in selected clinical trials [8, 9]. Recombinant factor FVIII and FIX products have shown sound hemostatic effects in terms of bleeding risk in hemophilia A or B [10]. FVIIa are very beneficial in the hemostatic treatment of patients with congenital coagulation factor defects and hematologic malignancies [11]. It has also been applied in topical dressings such as gel sponges or microporous polysaccharide spheres, thereby improving the overall hemostatic capacity of the dressing [12]. Wound healing is an essential process after hemostasis of trauma, which any of the above hemostatic modalities cannot significantly promote. Therefore, to deal with the problem of hemostasis and repair in an integrated manner, a biomaterial with early and rapid hemostasis and wound repair functions is urgently required.

There are a large number of reports related to hemostatic biomaterials research. Collagen and gelatin are important bioactive hemostatic materials with significant effects on further platelet activation. However, animal-derived collagen poses an immunogenic risk, and gelatin has limited effectiveness in controlling severe bleeding [13]. Plant fiber-derived oxidized regenerated cellulose (ORC) is an absorbable topical hemostatic agent widely used in surgical procedures for abdominal, oral, and intracranial hemostasis, etc. It can cause platelet aggregation and clotting through contact activation of platelets [14]. Surgicel™, designed and manufactured by Johnson and Johnson Medical, Inc., is the most widely used absorbable ORC hemostatic product. It enriches platelets, red blood cells, and other blood cells rapidly, motivating the secretion of coagulation factors to promote blood clotting. Furthermore, the low pH environment produced by the carboxyl groups on the molecules can effectively reduce the infections caused by certain pathogenic bacterial strains. However, the carboxyl groups in ORC will also form a local acidic environment in the wound, which is not conducive to wound healing. Its relatively poor degradability (the complete absorption requires 8 weeks) results in some clinical side effects. For example, there are reports that foreign body reactions occurred in patients with hypertensive cerebral hemorrhage after debridement with ORC [15–18]. Negatively charged uronic acid alginate hydrogels can bind to positively charged calcium ions to activate the coagulation cascade reaction [19]; alginate materials are also combined with other hemostatic materials, such as collagen, gelatin, oxidized cellulose, and chitosan, for the development of hemostatic and antibacterial composite hemostatic materials [19–21]. The electrostatic interaction of natural polysaccharide materials, chitin, and chitosan, with negatively charged red blood cell membranes, leads to agglutination of red blood cells to form hemostatic plugs [22–24]. It has also been reported to enhance primary hemostatic mechanisms, that is, to strengthen platelet adhesion, activation, and aggregation, and to adsorb fibrinogen from plasma to trigger complement activation [25]. All of the above biomaterials have shown varying degrees of hemostatic effects, whereas the following stage of tissue repair is not as effective. Cell migration, proliferation, deposition, and extracellular matrix formation are crucial in wound healing and recovery of skin tissue function. Meanwhile, they all depend on blood vessels to supply oxygen, nutrients, and signals to the cells. Therefore, the promotion of blood flow reconstruction is pivotal to enhance wound healing and skin function recovery [26].

To date, many techniques and dressings utilizing growth factors and drugs to promote wound vascularization have been researched. Losi et al*.* showed that in a model of hindlimb ischemia, simultaneous delivery of FGF-2 and VEGF-A increased the number of endothelial cells near the scaffold as well as blood perfusion in the hindlimb of rats after ischemic injury [27]. Platelet-derived growth factors (PDGFs) recruit cells to the wound site, stimulating wound angiogenesis. Electrospun nanofiber scaffolds carrying PDGF-BB and VEGF-A can enhance wound angiogenesis [28]. An electrospun nanofiber scaffold releasing FGF-2, EGF, VEGF-A, and PDGF-BB, composed of collagen and hyaluronic acid (HA), is proved to accelerate wound healing vascular maturation in a diabetic rat model [29]. Although wound healing agents based on growth factors facilitate wound vascularization and healing to a certain extent in clinical use, they have drawbacks such as high price, harsh transportation and storage conditions, short half-life, and uncontrollable release in vivo. By comparison, these defects can be overcome by biologically active drugs. The effect of simvastatin on diabetic mice has been investigated, given the pro-angiogenic properties of statins. Intraperitoneal injection of simvastatin for 3 and 6 days resulted in increased expression of VEGF-A protein and gene; after 12 days, improved wound healing and increased angiogenesis were observed [30]. Lingzhi Kong et al*.* suggested that bioglass acting synergistically with desferrioxamine (DFO) to promote vascular remobilization could boost chronic wound healing [31]. Hou et al*.* reported that diabetic wounds treated with DFO showed increased activity of HIF-1α and promoted the wound healing process [32]. All the active ingredients mentioned above contribute to vascularization and wound healing. Still, they cannot trigger the coagulation mechanism thus have any hemostatic effect in the early stage of trauma. Therefore, their combination with hemostatic biomaterials is expected to achieve the integration of hemostasis and repair. However, the simple combination of hemostatic materials and angiogenic drugs brings about the problem of drug burst release thus cannot guarantee the long-term stable promotion of wound vascularization. Therefore, the development of new biomaterials with the sustained-release effect of angiogenic drugs, early and rapid hemostasis, and long-term stable promotion of angiogenesis becomes the key to achieving the integration of hemostasis and repair.

Oxidized bacterial nanocellulose (OBNC) has the same chemical structural unit as ORC with more excellent properties, such as higher purity, the three-dimensional network structure of nanofibers, and ultra-high specific surface area, making it a promising biosynthesized biomaterial in the field of rapid hemostasis [14, 33–35]. In this study, bacterial nanocellulose (BNC) was produced by fermentation of *Komagataeibacter* *xylinus*. Then, by selective oxidation of hydroxyl groups to carboxyl groups through TEMPO, oxidized bacterial nanocellulose (OBNC) was obtained. OBNC was further grafted with DFO through an amide bond to achieve long-term slow release of the drug. OBNC facilitates coagulation at the early stage of trauma; meanwhile, slow release of DFO promotes long-term vascularization. Thus, a novel biomaterial with multiple functions of DFO slow-release effect, early rapid hemostasis, and long-term promotion of tissue repair was constructed. First, we investigated the microstructure, water absorption capacity and speed of BNC, OBNC, and OBNC-DFO. Secondly, we examined their hemocompatibility and coagulation effects in vitro, including hemolysis ratio, the adhesion and activation effects of platelets and erythrocytes, whole blood coagulation time, and plasma recalcification time. Next, to reveal the angiogenetic potential of DFO grafting at the cellular level in vitro, the effects of the materials on the proliferation and cell morphology of human umbilical vein endothelial cells (HUVECs) were investigated, and the differences in the concentration of HIF-1α secreted by HUVECs were compared. And then, rat tail amputation and liver trauma models were established, and the rapid hemostatic ability of the materials was evaluated by blood loss after treatments. Finally, the rat models of full-thickness skin injury were treated with different materials to observe the degree of wound healing at 1, 2, 3, and 4 weeks. HE and Masson staining were used to observe the inflammatory response and healing process; CD31 and a-SMA immunofluorescence staining was employed to monitor the level of neovascularization and mature blood vessels; CK14 was employed to evaluate the degree of epithelialization and the number of hair follicles; COL staining was used to determine the level of collagen deposition. The effects of OBNC-DFO on vascularization and repair of injured tissues were revealed at the animal level.

## Results and discussion

### Morphology, absorption, mechanical property and component analysis

The collagen hemostatic sponges (COL, trade name: Beiling, produced by Beijing Yierkang Bioengineering Development Center) used in clinical operating rooms have satisfactory hemostatic effects. In this study, these were used as control materials to evaluate the hemostatic properties of the constructed materials in micromorphology, water absorption properties, and coagulation experiments in vitro and vivo.

**Fig. 1:**
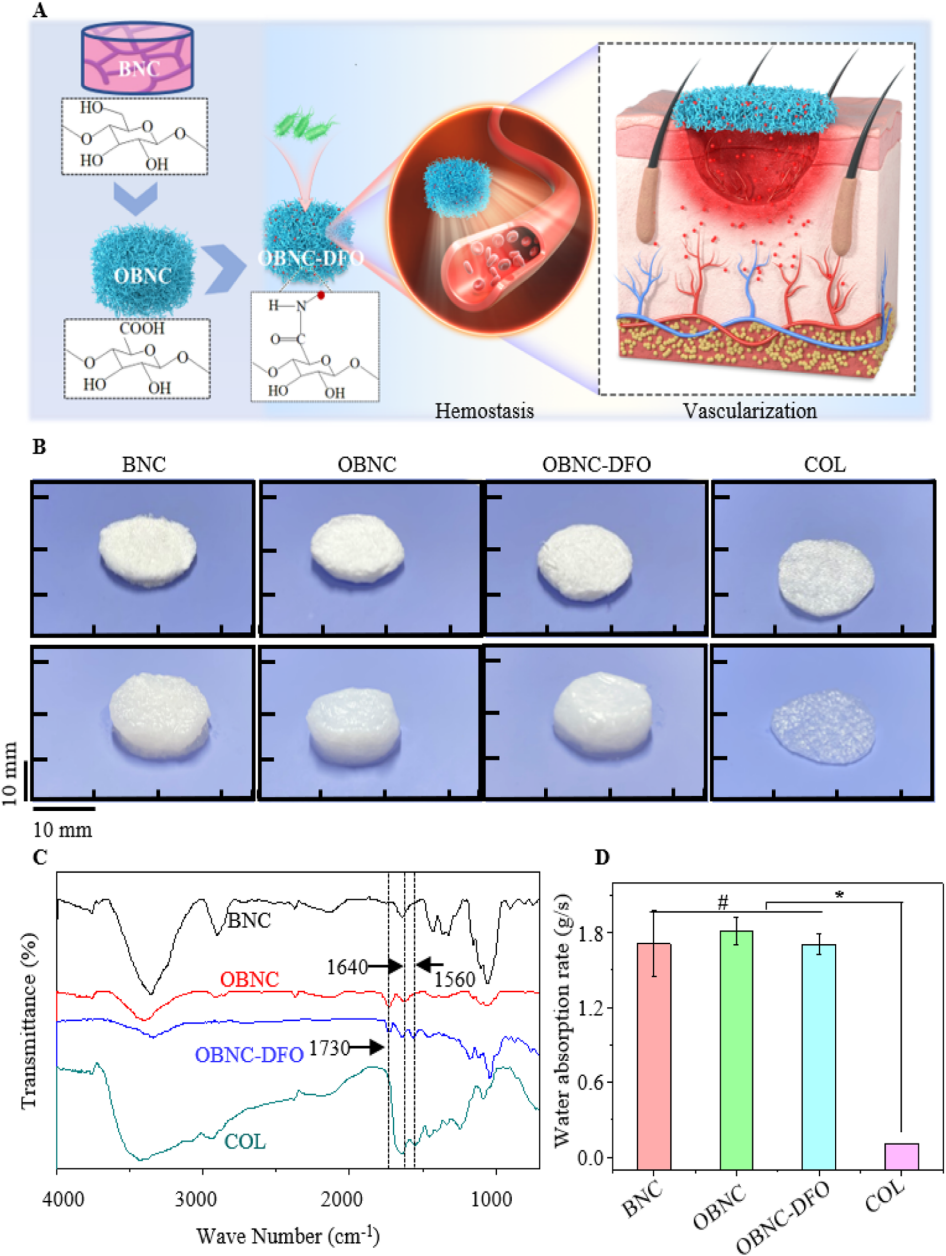
Schematic diagram of the preparation of OBNC and OBNC-DFO with BNC as raw material, and application for hemostasis and vascularization (**A**), macroscopic images before (in the first horizontal row) and after (in the second horizontal raw) water absorption (**B**), FTIR spectra (**C**), the water absorption rate (**D**) of BNC, OBNC, OBNC-DFO and COL. (*p < 0.01)

**Fig. 2:**
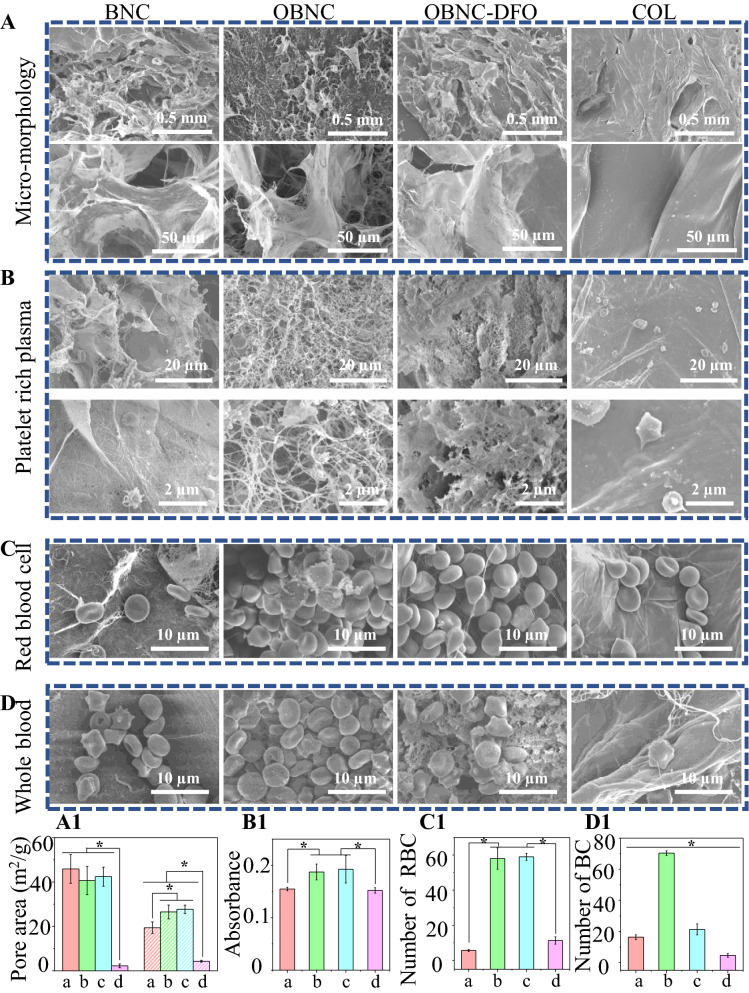
SEM images of the morphology of different samples under different magnification (**A**), pore area measured by BET on the left of A1 and mercury intrusion method on the right of A1, platelet adhesion observed by SEM after incubation with platelet-rich plasma (**B**), absorbance at 450 nm measured by CCK-8 assay (**B1**), red blood cell adhesion observed by SEM after incubation with red blood cells (**C**) and the number of red blood cells in the field of view (**C1**), blood cell adhesion observed by SEM after incubation with whole blood (**D**) and number of whole blood cells in the field of view (**D1**). The letter of a, b, c, d stands for sample of BNC, OBNC, OBNC-DFO and COL, respectively. (*p < 0.01)

From the macroscopic morphology, BNC, OBNC, and OBNC-DFO all exhibited extremely fluffy spongy shapes that could be compressed. The compression process of OBNC sponge was shown in the video of Additional file 1. The morphology after compression is shown in the top row of Fig. 1B, and the volume expands after water absorption is shown in the bottom row of Fig. 1B. Although named “sponge”, the collagen hemostatic material (COL) used in clinical practice is not easily compressible. It does not expand in volume after water absorption in the last column of Fig. 1B. The related results of the water absorption rates are shown in Fig. 1D. It can be seen that their absorption rates are approximately 1.71, 1.81, 1.70 and 0.11 g/s, respectively. There is no significant difference among the water absorption rate of BNC, OBNC and OBNC-DFO, but they are significantly faster than COL. The water absorption rates are closely related to the pore size and specific surface area. The micro-morphologies of BNC, OBNC, OBNC-DFO, and COL sponges are shown in Fig. 2A. The sponges of BNC origin (BNC, OBNC, and OBNC-DFO) have more pores than COL, further verified by the specific surface area measured by the nitrogen adsorption method. The BET results are shown in Fig. 2A1, where the surface areas of the BNC-derived sponges (BNC, OBNC, and OBNC-DFO) were approximately 45.903, 40.628, and 42.429 m^2^/g, respectively. However, the porosity of COL was only 2.262 m^2^/g. Total pore area measured by the mercury porosimeter were approximately19.417, 26.507, 27.751 and 4.315 m^2^/g. In both methods, total pore area of BNC-sourced materials (BNC, OBNC and OBNC-DFO) were significantly larger than that of COL. The absorption speeds of the BNC, OBNC, and OBNC-DFO sponge samples were much faster than that of COL, attributed to the larger porosity and specific surface area. However, the pore area of BNC-sourced material measured by the mercury intrusion method was significantly lower than that measured by the BET method for the same sample. The reason was that the test principles of the two methods were different. BET measures the specific surface area by the amount of nitrogen adsorption, which measures micropores, and the mercury intrusion method measures the specific surface area by the amount of mercury intrusion, and measures the macropores. Moreover, the mercury intrusion process was accompanied by the collapse of pores in the mercury intrusion method.

The tensile mechanical properties of BNC, OBNC, OBNC-DFO and COL sponges were shown in Table 1. The elongation at break of them were about 17.18%, 11.41%, 12.55% and 12.98%. The elongation of OBNC and OBNC-DFO sponges were significantly lower than BNC, which indicated that ductility reduced after oxidation, but had no significant difference with COL. Tensile strength of them were about 20.31, 40.07, 40.31 and 248.51 kPa, and Young’s modulus of them were about 1.78, 2.70, 2.73 and 21.36 kPa. The results showed that the tensile strength and Young’s modulus of COL was much higher than others. Although the mechanical properties of sponges derived from BNC were not as good as COL, it should be more comfortable for the wound due to its softness.

**Table 1:** Mechanical tensile properties

	BNC	OBNC	OBNC-DFO	COL
Elongation at break (%)	17.18 ± 1.20^*^	11.41 ± 0.90	12.55 ± 1.36	12.98 ± 0.37
Tensile strength (kPa)	20.31 ± 6.92^*^	40.07 ± 2.63	40.32 ± 2.88	248.51 ± 30.16^**^
Young’s modulus (kPa)	1.78 ± 0.62^*^	2.70 ± 0.88	2.73 ± 1.01	21.36 ± 2.71^**^

The labels (^*^ or ^**^) in the chart mean that significant difference (p < 0.01) was detected among the groups in the same row (n = 3)

Based on the FTIR spectra, OBNC and OBNC-DFO displayed characteristic absorption peaks at 1730 cm^−1^, corresponding to the C = O stretching vibration of carboxyl groups. OBNC-DFO possessed two characteristic absorption peaks at 1640 and 1560 cm^−1^, corresponding to the amide I and amide II of the peptide group from DFO, confirming the successful grafting of DFO.

### In vitro hemostatic assay

**Fig. 3:**
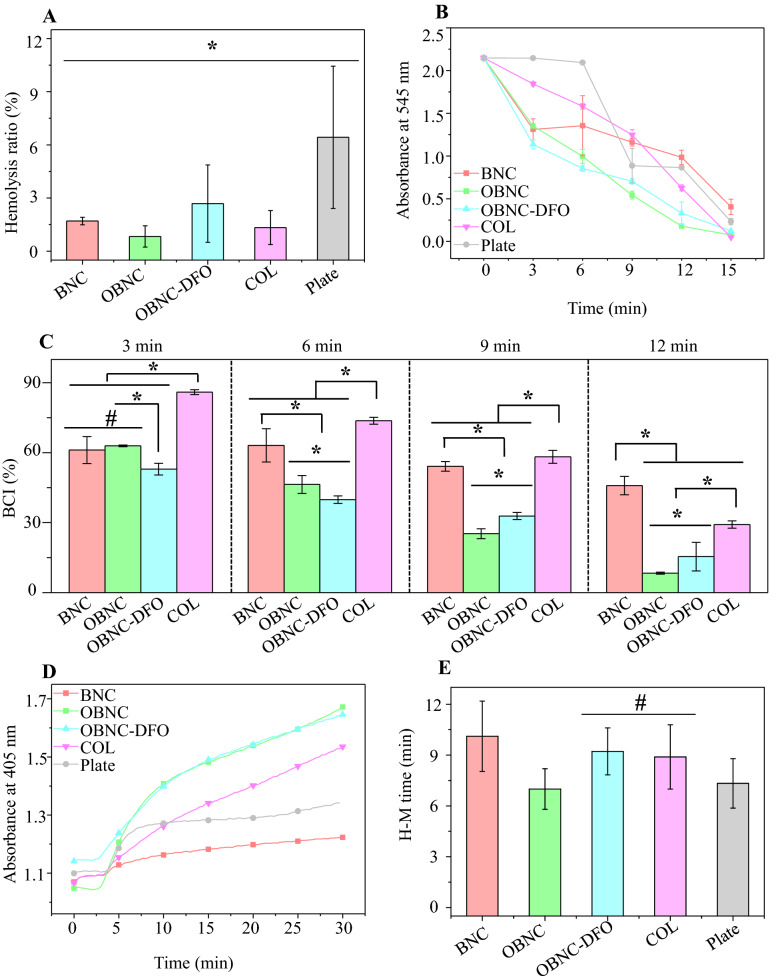
Hemocompatibility and coagulation assay in vitro, hemolysis ratio (**A**), whole blood coagulation process (**B**), blood coagulation index (BCI) after whole blood touching with samples of 3, 6, 9, 12 min (**C**). plasma recalcitrance kinetic curve (**D**) and plasma recalcitrance time (**E**) of BNC, OBNC, OBNC-DFO, and COL, blank wells of plate (Plate) were used as a control. (*p < 0.01, #p > 0.05)

The hemolysis ratio demonstrates the hemocompatibility of biomaterials. After incubation with BNC, OBNC, OBNC-DFO, and COL sponges, the results are shown in Fig. 3A. The hemolysis ratios of each sample are 1.78%, 0.83%, 2.69%, and 1.34%, which are all lower than 5%, indicating that they have excellent hemocompatibility.

Wound dressings with rapid hemostatic capacity are crucial to decrease trauma-related mortality by reducing blood loss in the first place. Platelets are one of the most important mediators of hemostasis and thrombosis. After incubation of platelet-rich plasma with the material, platelet adhesion could be observed by FE-SEM. Platelet pseudopods caused by activation were observed on the surface of all samples, as shown in Fig. 2B. It has been reported that activated and adhered platelets could be assayed quantitatively by the absorbance values in the CCK-8 experiments, with higher absorbance values implying more adherent platelets. In Fig. 1b, the absorbance values of the OBNC and OBNC-DFO groups showed no significant differences between each other. Still, both of them were higher than the BNC and COL groups, indicating more activated and adhered platelets.

References suggest that erythrocytes play an important role in hemostasis and wound healing through various mechanisms. Figure 2 C and C1 present the morphology and statistical results of adhered erythrocytes, which are observed on the surface of different materials by SEM. The higher number of adhered erythrocytes in OBNC and OBNC-DFO indicates that oxidized bacterial nanocellulose promotes the adhesion of erythrocytes.

The dynamic experiment of whole blood clotting reflects the complicated influence between biomaterials and blood due to the complex composition of blood. The dynamic process of whole blood clotting is shown in Fig. 3B. During the experiment, a lower absorbance value at 545 nm indicates more clarified supernatant, more blood clots formed on the material's surface, and a more significant coagulation effect. In order to reflect the coagulation effect more intuitively, the blood coagulation index (BCI) for different time periods of contact with blood was recorded, which was shown in Fig. 3C. The lower the BCI, the better the coagulation effect. It can be seen from the dynamic curve of the whole blood coagulation, blood samples incubated with OBNC and OBNC-DFO sponges showed lower absorbance values than those incubated with BNC (except that there was no significant difference between BNC and OBNC at 3 min), the corresponding BCI were approximately 61.13%, 62.95%, 52.96% and 85.96% at 3 min, 63.13%, 46.39%, 39.87% and 73.71% at 6 min, 54.17%, 25.25%, 32.85%. and 58.23% at 9 min, 45.89%, 8.35%, 15.43% and 29.21 at 12 min. It demonstrating that oxidized bacterial nanocellulose is superior in promoting whole blood clotting. It was worth noting that the coagulation effect of OBNC-DFO was significantly better than OBNC at 3 and 6 min. The possible reason was that the trace amount of DFO in OBNC-DFO chelated the iron ions in the blood which cause blood clotting. Clinically used COL incubations were significantly less effective at clotting in the first 15 min than oxidized cellulose (OBNC and OBNC-DFO). Whole blood samples incubated with all materials were almost completely clotted at 15 min with absorbance values close to 0. The statistics of adhered blood cells are shown in Fig. 2D and D1. Remarkably more blood cells adhered to oxidized bacterial nanocellulose (OBNC and OBNC-DFO) than to BNC and COL. What is noteworthy is that a large amount of other material (aggregated plasma proteins and platelets) is adhered to the surface of OBNC-DFO along with blood cells. It could not be concluded that the number of adherent blood cells on the surface of OBNC-DFO was less than that of OBNC with lots of fibrous material wrapped around on the former, which caused statistical difficulties.

Plasma recalcification kinetic analysis using PPP, which detects changes in absorbance influenced by the addition of Ca^2+^ to the sample, is commonly used to assess the activation of endogenous coagulation pathways. The time for the absorbance to reach half of the maximum value is called the plasma recalcification time (H-M time). As shown in Fig. 3D and E, the absorbance values of the samples incubated with OBNC and OBNC-DFO were significantly higher than those incubated with BNC and COL. The H-M time of OBNC was substantially lower than that of BNC, indicating that oxidized bacterial nanocellulose activates the endogenous coagulation pathway in a shorter time. Although previous studies have also tested the plasma recalcification time of cellulose-based materials, the data from different batches could not be compared horizontally. The degree of blood activation was different due to various blood sources; the concentration of Ca^2+^ added for better observation, on the other hand, is also likely to vary.

### In vitro HUVECs assay

**Fig. 4:**
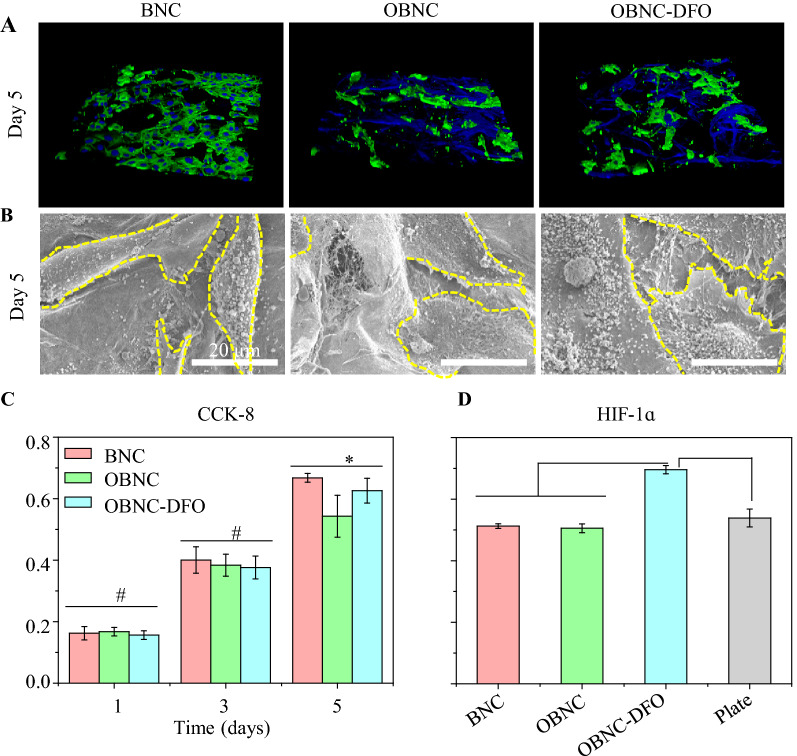
In vitro HUVECs assay. Fluorescence staining results of FITC-labeled phalloidine (green) and DAPI (blue) observed by confocal microscopy (**A**), SEM images of individual cell morphology (**B**), absorbance at 450 nm after 1, 3, and 5 days as measured by CCK-8 assay (**C**) and HIF-1α protein concentration in cell supernatant measured by ELISA kit after 5 days of culture of HUVECs (**D**). (*p < 0.01, #p > 0.05)

CCK-8 assay was used to evaluate the proliferation of HUVECs quantitatively on BNC, OBNC and OBNC-DFO at 1, 3 and 5 days, respectively. The results obtained are displayed in Fig. 4C. They showed that cells on the surfaces of the three materials continued to proliferate during 5 days of incubation, proving that all materials (BNC, OBNC and OBNC-DFO) were free of cytotoxicity. For the first 3 days of incubation, no significant differences were observed on the surface of the samples. Differences appeared on day 5 when the number of cells on the surface of BNC exceeded that of OBNC, indicating that oxidized bacterial nanocellulose inhibited cell proliferation. It has been reported that OBNC with carboxyl group creates an acidic environment, which suppresses cell proliferation, as well as a certain inhibitory effect on bacteria according to the previous reports [3, 36]. However, the number of cells on the surface of OBNC-DFO grafted with DFO was remarkably higher than that of OBNC, illustrating that DFO at this concentration could promote the proliferation of HUVECs to some extent.

The fluorescence staining results are shown in Fig. 4A. The cytoskeletons were stained green by FITC-labeled phalloidine, and the nuclei were stained blue by DAPI. OBNC and OBNC-DFO were also stained blue by DAPI, but it did not affect the observation of the cells on the sponges. It is clear that the cells on BNC are significantly more than on OBNC and OBNC-DFO, which is consistent with the results of CCK-8 assay. The morphology of individual cells was clearly observed by FE-SEM. The morphology of cells on BNC was long spindle-shaped, which was markedly different from the cells spreading on OBNC and OBNC-DFO.

HIF-1α protein expression is shown in Fig. 4D. Cells on the surface of the OBNC-DFO group secreted more HIF-1α due to the graft and release of DFO. HIF-1α facilitates the secretion of vascular endothelial growth factor and promotes the proliferation of HUVECs, which is why at day 5, the number of cells on the surface of OBNC-DFO was higher than that of OBNC.

### In vivo hemostatic assay

**Fig. 5:**
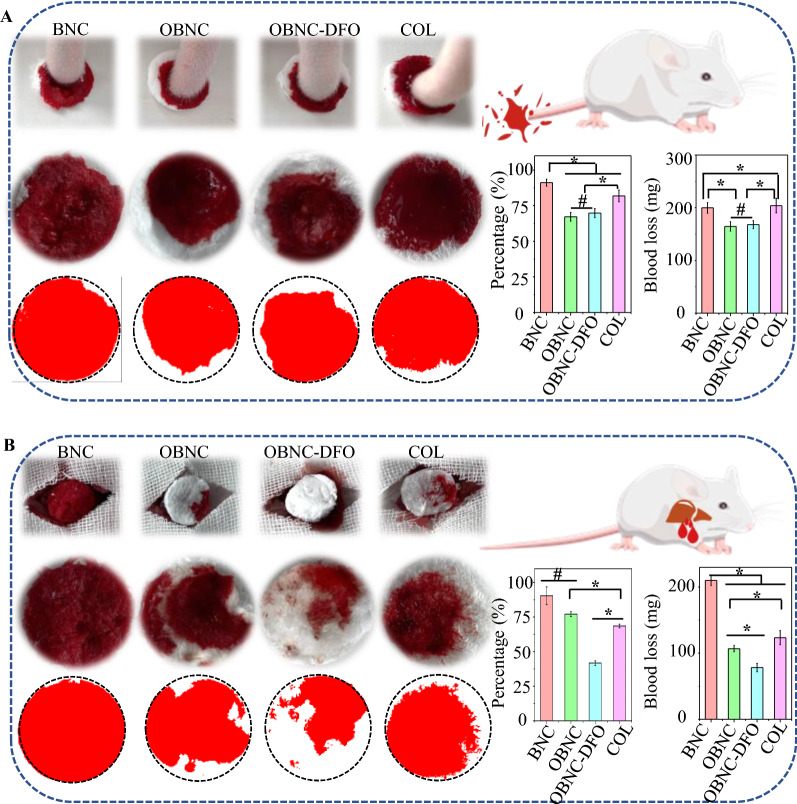
In vivo evaluation of the hemostatic capacity of the different sponges (n = 3). In the rat tail amputation model (**A**) and liver trauma model (**B**), gross images, area of absorbed blood (with schematic diagram), blood infiltration percentage calculated by image J, and blood loss. (*p < 0.01, #p > 0.05)

The animal hemostatic effect of sponges (BNC, OBNC, OBNC-DFO, and COL) was further investigated in the rat tail amputation model (Fig. 5A) and liver trauma model (Fig. 5B) by measuring the surface blood infiltration area of the sponge as well as the amount of blood loss. In the rat tail amputation model, the percentage of surface blood infiltration area was approximately 91.14%, 67.14%, 69.81%, and 81.78%, and the blood loss was about 190.47, 160.15, 159.46, and 200.26 mg, respectively. From the above results, OBNC and OBNC-DFO did not differ notably from each other in surface blood infiltration area and blood loss, and both of them are lower than BNC and COL. In the liver trauma model, the percentage of surface blood infiltration area was approximately 90.46%, 77.14%, 41.72%, and 68.59%, and the blood loss was about 200.42, 96.28, 80.44, and 110.37 mg, respectively. The hemostatic effect was listed in the following order: OBNC-DFO > OBNC > COL > BNC. It shows that the results of the surface blood infiltration area coincided with blood loss. It suggests that the in vivo hemostatic effect of oxidized cellulose (OBNC and OBNC-DFO) was significantly superior to that of BNC and the clinically used hemostatic material collagen hemostatic sponge (COL).

### Wound healing evaluation

**Fig. 6:**
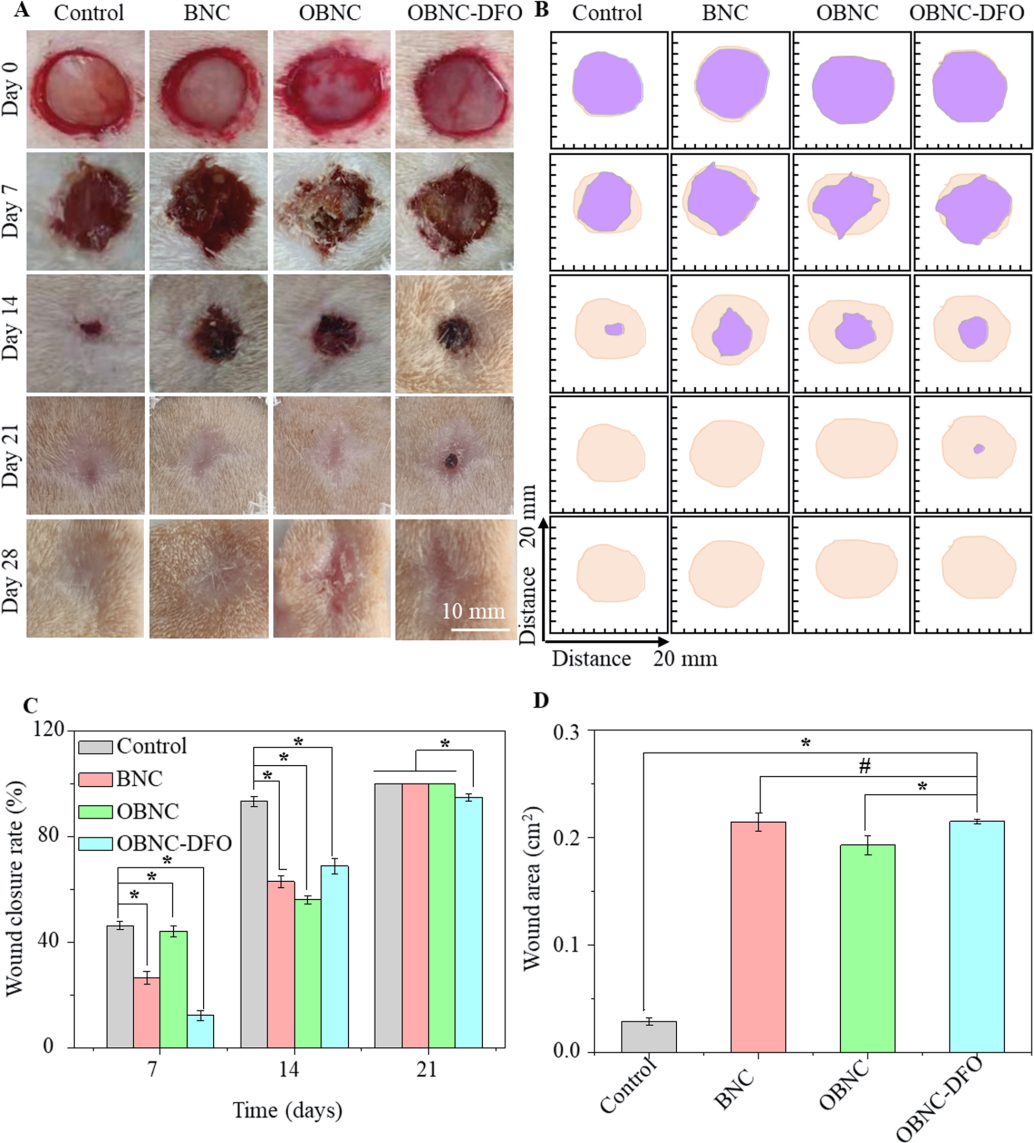
Wound healing evaluation in rats with full-thickness wounds. Images of wound healing of BNC, OBNC, OBNC-DFO and control groups on days 0, 7, 14, 21, and 28 (**A**), and schematic diagram of wound healing area (**B**), skin color and purple color indicating wound area at initial and different time points, respectively. Wound closure rate relative to the initial wound at different time points (**C**) and wound area at 21 days (**D**) measured by image J. (*p < 0.01, #p > 0.05)

To evaluate the effect of hemostatic materials on wound healing, we applied sample materials (BNC, OBNC, and OBNC-DFO) to rats with full-thickness wounds. Wounds in the control group were left untreated. Figure 6A shows the gross morphological results of the wound surfaces on days 0, 7, 14, 21, and 28. No significant infection was observed at each time point after surgery, with the wound area decreasing with time. The macroscopic morphology of the untreated control group at day 7 showed incomplete crusting, whereas both OBNC and OBNC-DFO sponges exhibited complete crusting and appeared drier. The reason is that the sponges absorbed secretions to remain dry. The rate of wound closure was evaluated based on the wound area at initial and different time points (Fig. 6C). From the results, the wound closure rate of the oxidized cellulose sponge was not as good as that of the control group. Combined with the cell proliferation results (Fig. 4), it can be perceived that oxidized cellulose does not effectively enhance cell proliferation in the absence of other active ingredients. Therefore, it is reasonable that it did not promote wound healing.

However, wound healing is a complex process. It involves spatial and temporal synchronization of various cell types who play specific roles in the hemostasis, inflammation, growth, re-epithelialization, and remodeling phases [2]. The skin is a functional tissue with many appendages such as hairs, sweat glands, capillaries, etc. Slow surface wound closure indicates a slower rate of epithelialization rather than insufficient repair of the skin. An important aspect of wound healing and repair is creating a new vascular system through angiogenesis, which provides adequate oxygen, nutrients and signals for cell migration, proliferation, deposition, and extracellular matrix formation [37].

**Fig. 7:**
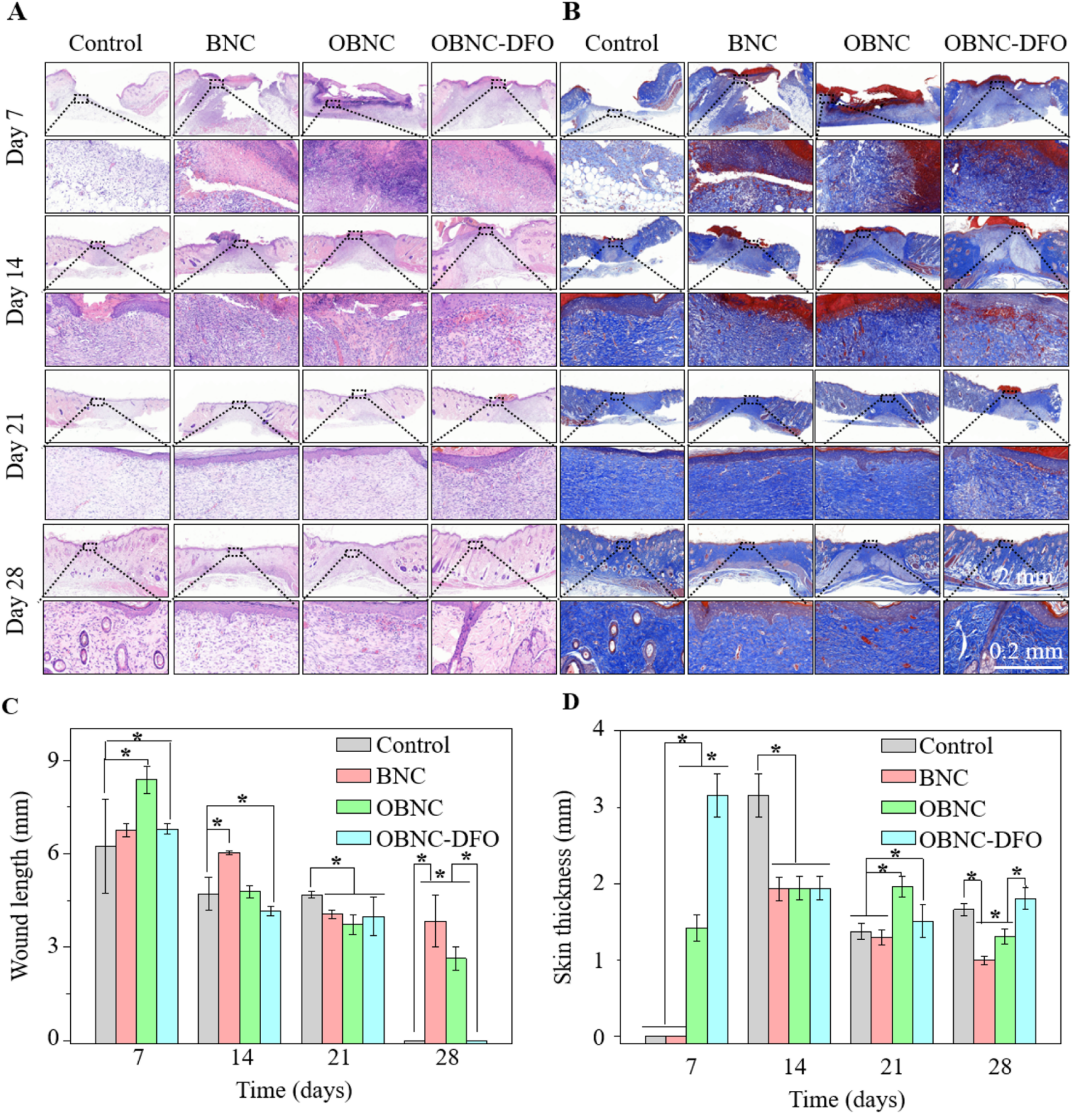
Immunohistochemical results at different time points (on days 7, 14, 21, 28). Images of HE staining (**A**) and MASSON staining (**B**), wound healing length (**C**) and skin thickness at different time points (**D**), (*p < 0.01)

Masson trichrome staining was performed on the skin derived from the wound site, as shown in Fig. 7A and B, to assess the inflammatory response and collagen deposition during the healing process. According to HE staining, the skin tissue with regenerating appendages was considered to start repairing, and the length of the unrepaired wound was recorded, as shown in Fig. 7C. The wound thickness was also measured based on HE and MASSON staining, and the results are shown in Fig. 7D. In the first 14 days after surgery, gross observation noticed no significant inflammatory reaction in all specimens. Nevertheless, the HE staining results showed that the inflammatory response was slightly stronger in the OBNC and OBNC-DFO sponge groups than in the BNC and control groups. An appropriate level of inflammatory response would be beneficial to the revascularization [38].

**Fig. 8:**
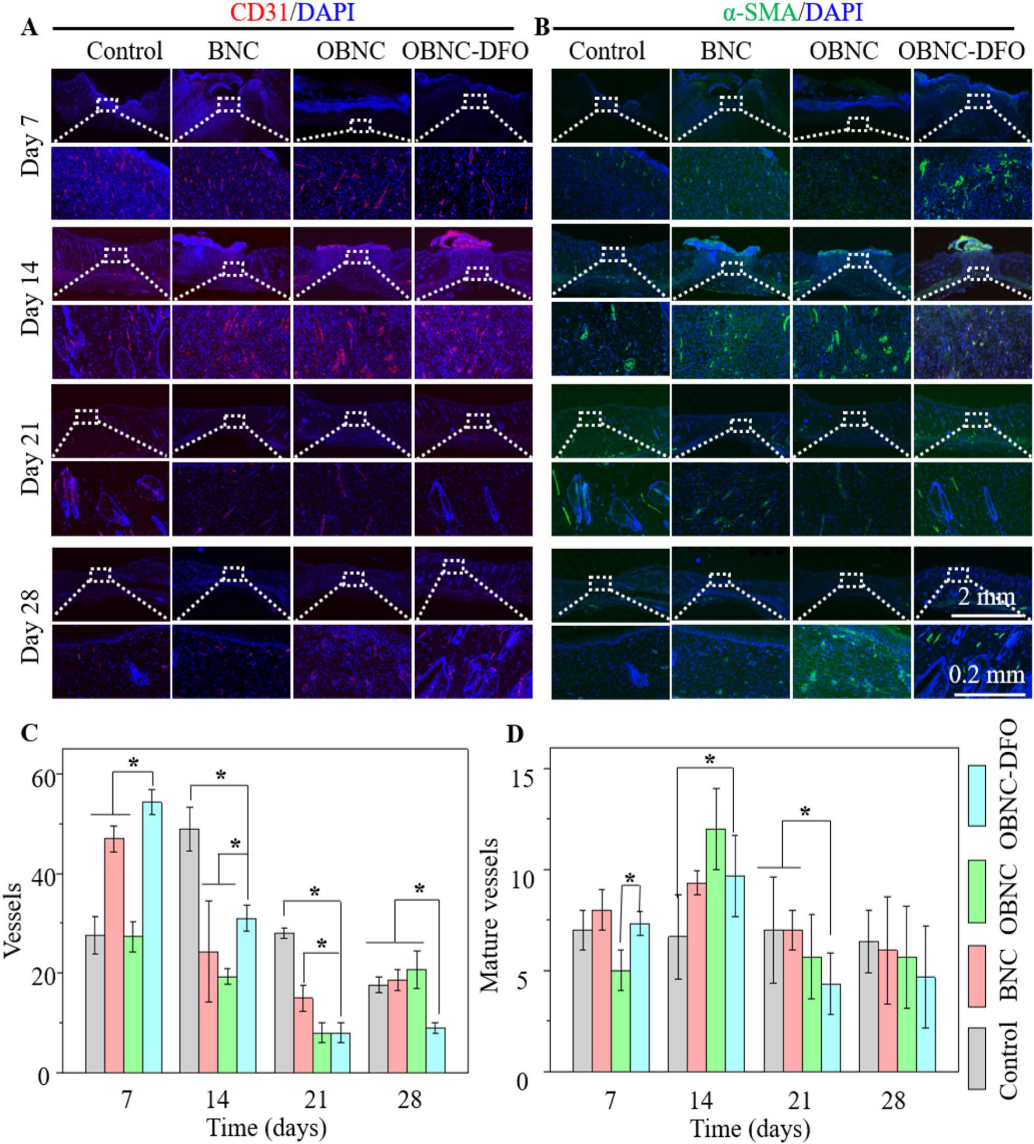
Immunofluorescence staining results at different time points (on days 7, 14, 21, 28) of control, BNC, OBNC or OBNC-DFO groups. Images of CD31 staining (**A**), α-SMA staining (**B**), number of newly formed vessels according to CD31 results (**C**), number of mature vessels according to α-SMA results (**D**), (*p < 0.01)

CD31 and α-SMA stainings, showing neovascularization and mature blood vessels, respectively, were displayed in Fig. 8A and B. The number of neovascularization and mature blood vessels in the 40 × objective view was counted (Fig. 8C and D). The neovascularization in the normal dermal tissue of the skin is at a comparatively low level. Once the skin is damaged, an appropriate inflammatory response induces neovascularization to provide oxygen and nutrients to tissues in the wound healing stage. After skin repair is completed, nonessential vessels degenerate, and only essential vessels are retained. In the current study, the OBNC-DFO group showed the highest level of neovascularization on day 7, followed by a decreasing trend, indicating that OBNC-DFO can promote the induction of neovascularization during the first stage wound healing. It is mainly attributed to the released DFO enhancing the secretion of HIF-1a, which facilitates neovascularization. On day 21, the wound healing and the extracellular matrix remodeling phases are almost completed in the OBNC-DFO group. The control group had the highest number of vessels on days 14 and 21. It failed to promote angiogenesis at an early stage, thus delaying tissue regeneration.

**Fig. 9:**
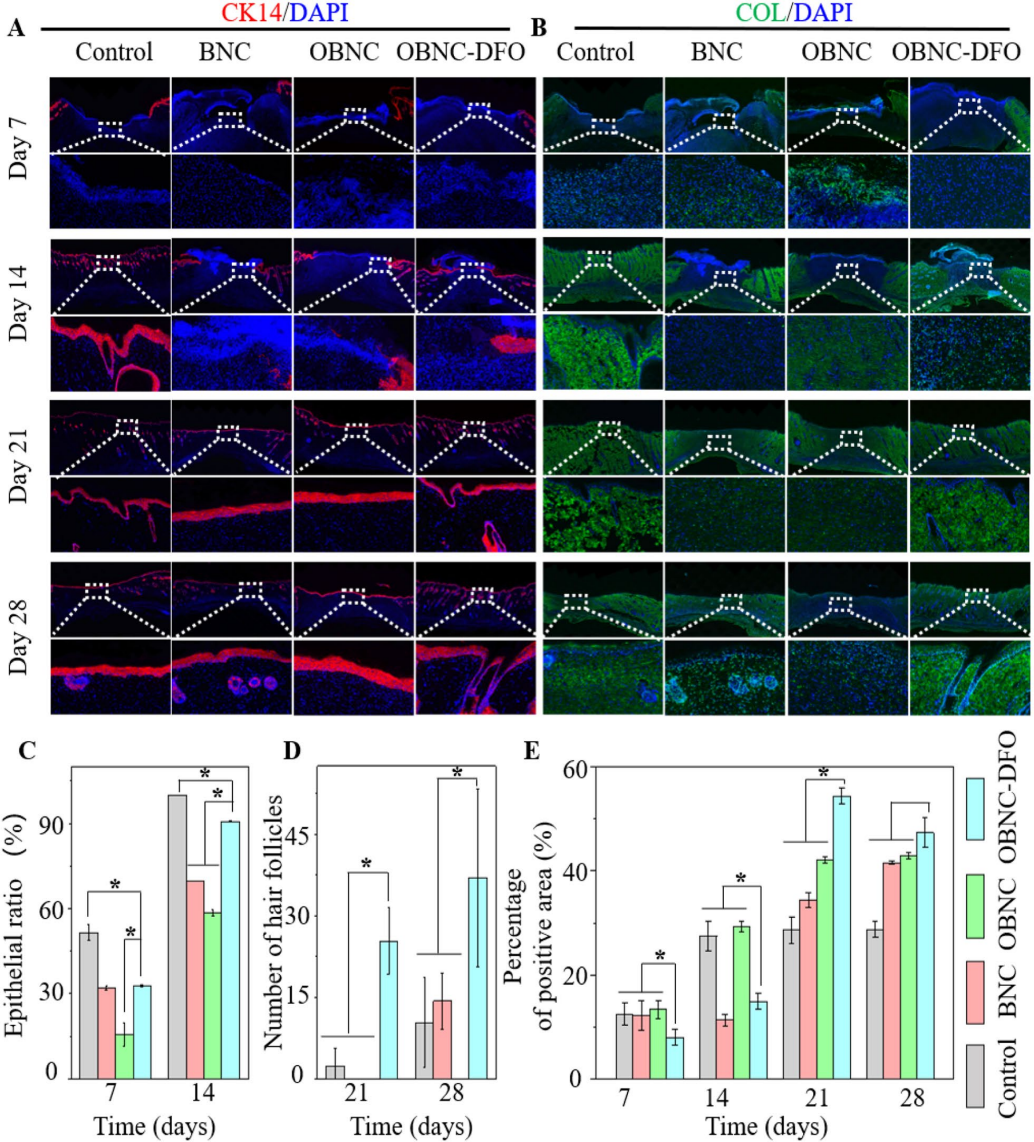
Immunofluorescence analysis of epithelialization and collagen deposition of control, BNC, OBNC or OBNC-DFO groups. Images of CK14 staining (**A**) and of type I collagen staining (**B**). The degree of epithelialization on days 7 and 14 (**C**) and the number of hair follicles (magnification: 40×) on days 21 and 28 (**D**) according to CK14 results. The percentage of type I collagen-positive area (**E**) was calculated by image J

CK14 is a member of the keratin family and is expressed mainly in hair and epithelial cells. The skin repair process is accompanied by collagen deposition, which is closely related to the repair mechanism. CK 14 and collagen I (COL) stainings were performed, and the results are shown in Fig. 9A and B. All samples were completely epithelialized on day 21. In the results of the first 14 days, it can be seen that the epithelialization rates of experimental groups (BNC, OBNC, and OBNC-DFO) were significantly slower than the control group, revealing that the material treatment was not favorable for epithelialization. However, on 21 and 28 days, the number of hair follicles in the ONC-DFO group was considerably higher than that of the control group. Therefore, it can be concluded that the rate of skin appendages regeneration in the OBNC-DFO group was markedly higher than that of the control group, which was more favorable for skin repair. Through the statistical analysis, the percentage of collagen I deposition showed an increasing trend over time in all groups after 14 days, and the OBNC-DFO treated group reached the highest rate of type I collagen-positive area on day 21. By correlating the pathological findings with the staining of neovascularization and hair follicles, the OBNC-DFO treated group exhibited a positive effect on tissue repair by favoring early angiogenesis, promoting collagen deposition and remodeling, as well as facilitating skin appendages regeneration.

## Conclusions

In this study, we designed a hemostatic sponge with multiple functions of immediate hemostatic effect and promotion of wound vascularization. It was constructed by fermentation of *Komagataeibacter* *xylinus* combined with TEMPO oxidation to obtain oxidized bacterial nanocellulose (OBNC), and then angiogenetic drug desferrioxamine (DFO) was grafted through an amide bond.

This hemostatic sponge has comparatively high porosity and specific surface area, which has excellent water absorption capacity and can rapidly absorb blood to promote clot formation. In addition, the released DFO can promote the secretion of HIF-1α from HUVECs, which is beneficial to the reconstruction of blood flow, thus achieving rapid hemostasis and vascularization in injured tissue. In conclusion, this OBNC-DFO hemostatic sponge can rapidly activate coagulation pathways and skin repair process, making it a highly promising biomaterial for integrated hemostasis and pro-regenerative repair.

## Materials and methods

### Materials

All the chemical regents were purchased from the Sinopharm Chemical Reagent Co., Ltd (Shanghai, China) unless specifically stated. Human umbilical vein endothelial cells (HUVECs) were purchased from institute of biochemistry and cell biology, Chinese Academy of Sciences (Shanghai, China). Fetal bovine serum (FBS), dulbecco’s modified eagle medium (DMEM) high glucose culture medium, 10,000 U/mL penicillin, 10,000 μg/mL streptomycin, and trypsin-ethylene diamine tetraacetic acid were purchased from Life Technologies Corporation (New York, USA). Cell counting kit 8 (CCK-8) was purchased from Beyotime (Shanghai, China). Fluorescein isothiocyanate (FITC)-labelled phalloidin and 4′,6-diamidino-2-phenylindole (DAPI) were purchased from Yeasen Biotechnology Co., Ltd (Shanghai, China). Deferoxamine (DFO) was purchased from Sigma-Aldrich (Shanghai, China). Clean Sprague Dawley rats (200 g) were purchased from SLAC (Shanghai, China). Deferoxamine mesylate salt (DFO), chemical name is N′-[5-[[4-[[5-(Acetylhydroxyamino)pentyl]amino]-1,4-dioxobutyl] hydroxyamino]pentyl]-N-(5-aminopentyl)-N-hydroxysuccinamide), was purchased from Sigma-Aldrich (Shanghai, China).

1. **The preparation of BNC short fibers**
  The microbial fermentation generated the hydrogel-liked BNC membrane. Specifically, *Komagataeibacter* *xylinus* (*K*. *xylinus*, China General Microbiological Culture Collection Center Number. 1186) was cultured on the fermentation medium (100 g/L D- fructose, 5 g/L peptones, 3 g/L yeast extract, pH 5.0) for 7 days at 30 ℃. After that, the BNC membrane was obtained on the gas–liquid interface. Then, the BNC membrane was boiled in the 1% NaOH solution (w/v) for 4 h at 80 °C to lyse and remove bacteria and repeatedly washed with ultra-pure water until the pH was neutral. After that, the membrane was autoclaved at 121 °C for 30 min with substantial volumes of ultrapure water and repeated for 5 times in order to eliminate the endotoxin, ensuring the content of endotoxin was lower than the implantation standard of the third type of implantable device according to the United States Pharmacopoeia (USP), 2011, Chapters 85 and 161. Then, the BNC membrane was cut into small pieces and processed in the homogenizer (IKA T-25, Germany) for 15 min at 15,000 rpm with ultra-pure water to obtain the BNC emulsion. Finally, after freeze-drying, the BNC short fibers were obtained.
2. **The preparation of BNC, OBNC and OBNC-DFO sponge**
  BNC sponge of 1% (w/v) was prepared by quantifying BNC short fibers, resuspending and freeze-drying. Oxidized BNC (OBNC) was synthesized by the reported method of TEMPO selective oxidation. Concisely, 0.1 g dried BNC short fibers were resuspended in 30 mL sodium phosphate buffer (0.05 M, pH 6.86). Then 0.01 mmol TEMPO and 1.7 mmol NaClO_2_ were added into the BNC suspension. Next, 0.2 mL NaClO was added into 10 mL sodium phosphate buffer (0.05 M, pH 6.86), and mixed with the BNC suspension. The suspension was constantly stirred at 50 °C for 48 h. After that, the product was washed with ultra-pure water three times and freeze-dried to obtain the OBNC with unknown concentration. Finally, the obtained OBNC was weighted, resuspended, and freeze-dried to prepare the 1% OBNC sponge (w/v). According to reports, 2 μM DFO can promote the proliferation of HUVEC. Therefore, 2 µM DFO was used to resuspend OBNC (1%, w/v). After freezing drying, the OBNC-DFO sponge was prepared.
3. **The investigation of sponges**
  The general views of sponges were recorded by the digital camera when the sponges were compressed and swollen. The attenuated total reflection-fourier transform infrared (ATR-FTIR) explored the chemical structure of sponges after freezing drying. The water absorption performance of sponges was investigated as the following descriptions. After freezing drying, the sponges were weighted (the weights were denoted as Wdry = 0.02 g, quantitative to approximate 0.02 g) and immersed into the deionized water. At fixed time points (10, 20, 30, 60, 120, 180, and 240 s), the sponges were wiped and weighted (the weights were denoted as Wwet). When the weights of sponges were unchanged, the duration was recorded as the water absorption time (T). The water absorption rate was calculated as the following formulation: water absorption rate (g/s) = (Wwet-0.02)/T. The morphologies of sponges were observed by the field emission scanning electron microscope (FE-SEM, Sirion 200, US) after freezing drying and sprayed gold treatments. After freezing drying, the total pore area of sponges was analyzed by the surface area and porosity analyzer (Autosorb-IQ3BET, USA) and automatic mercury porosimeter (Micromeritics V 9620, USA) at 30,000 psia. Mechanical tensile properties of sponges (BNC, OBNC, OBNC-DFO and COL) were conducted by dynamic thermomechanical analyzer (DMA Q850, USA), and Young’s modulus was calculated according to the stress–strain curve.
4. **The investigation of coagulation performance in vitro**
  The commercial hemostatic collagen sponges (COL, commercial name: Beiling, Beijiang) were used as a control in vitro and in vivo coagulation experiments. To explore the coagulation performance in vitro, the sponges were evaluated in four parameters [39], platelet activation and adhesion test, hemolysis rate, whole blood coagulation test, and plasma re-calcification kinetic analysis. Specifically, sponges (BNC, OBNC, OBNC-DFO, COL) were sterilized by ultraviolet radiation for 24 h in the 24 well-plates. The investigation of coagulation performance in vitro was conducted in the 24 well-plates. Therefore, blank wells of plate were used as a control in this experiment. Fresh blood samples were isolated from the heart of white New Zealand rabbits with the addition of 3.2% (w/v) sodium citrate to prevent blood clotting. All the blood samples were stored at 4 ℃ before experiments. 10 mL whole blood was centrifuged at 100 g for 10 min. The upper suspension was platelet-rich plasma (PRP) that was used in the platelet adhesion test. The lower precipitation was adjusted to 10 mL with saline solution to obtain the red blood cell solution applied in the hemolysis rate and red blood cell adhesion test. Additionally, the whole blood was centrifuged at 3000 g for 5 min to isolate the platelet-poor plasma (PPP) for the plasma re-calcification kinetic analysis.
  *The platelet activation and adhesion test* PRP of 500 μL was added on the surfaces of sponges. Then sponges were incubated at 37℃ for 2 h. After that, sponges were washed with saline solution three times to remove the free platelet. Afterward, sponges were incubated with the mixture of 50 μL cell counting kit (CCK)-8 and 450 μL saline solution for 1 h at 37 ℃. The microplate reader evaluated the absorbance of suspension at 450 nm. In addition, the sponges were fixed at 2.5% glutaraldehyde for 4 h, followed by the gradient ethanol (25%, 50%, 75%, 95%, and 100%) dehydration treatment. Then sponges were processed with critical point drying and observed by the FE-SEM to evaluate the activation and adhesion of platelet.
  *Hemolysis rate* red blood cell solution of 120 μL was added to the surface of each sample (including negative and positive control). After 1 h’s incubation at 37 °C, saline solution of 2 mL was gently added into each sponge except that positive control was added with 2 mL sterile water, followed by centrifugation at 660 *g* for 5 min. Finally, suspension of 100 μLwas transferred into 96 wells plate, and the microplate reader measured the absorbance at 550 nm. The hemolysis rate was calculated as the formulation: Hemolysis rate (%) = (ODt − ODn)/(ODp − ODn) × 100, ODt: the absorbance of sponges, ODn: the absorbance of negative control, ODp: the absorbance of positive control. After the measurement on the microplate reader, these sponges were washed with saline solution and fixed with the 2.5% glutaraldehyde for 4 h, followed by the gradient ethanol (25%, 50%, 75%, 95%, and 100%) dehydration treatment. Then ethanol was replaced by the tert-butanol. After freeze-drying, the adhesive red blood cells on the sponges’ surface were observed and counted by the FE-SEM.
  *Whole blood coagulation test* CaCl_2_ (0.025 M) solution of 500 μL was mixed with 5 mL whole blood to active blood and stimulate the blood clotting. Then, activated whole blood of 100 μL was added to each sponge (blank well of 24-well plate was used as a control) followed by incubation at 37 ℃ for 0, 3, 6, 9, 12, and 15 min. At each timepoint, sterile water of 2.5 mL was added to each sample for 5 min of co-culturing. After gently mixing, suspension of 200 μL from each sample was transferred to 96 wells plate. The microplate reader measured the absorbance at 550 nm to describe absorbance changes over time for the simulation of the whole blood coagulation kinetic curve. Blood coagulation index (BCI) at different timepoint (3, 6, 9, 12 min) was calculated according BCI = 100* A_sample_/A_control_, which A_sample_ and A_control_ mean absorbance of sample and control at different timepoint. Afterward, the sponges were cleaned and treated as the processes mentioned above in the *Hemolysis rate* experiments. Finally, the adherent blood cells on the surface of sponges were counted and observed by the FE-SEM.
  *Plasma re-calcification kinetic analysis* PPP of 500 μL was added to each sponge, and PPP without any sponge was regarded as the negative control. After 1 h’s incubation at 37 ℃, suspension of 100 μL was transferred to 96 wells-plate with the addition of 100 μL CaCl_2_ (0.005 M) solution except that 100 μL saline solution was added into the negative control. The absorbance was measured at 405 nm for every 30 s, and the measurement lasted for 30 min to record the change of absorbance over time for describing the plasma re-calcification kinetic curve. Plasma re-calcification time means the time that the absorbance arrives at half of the maximum absorbance (denoted as the H-M time).
5. **In vitro cell experiments**
  Sponges (BNC, OBNC, and OBNC-DFO) were sterilized by ultraviolet radiation at 24 wells plate. Then 400 μL cell suspension consisting of 10% fetal bovine serum (FBS), 1% penicillin–streptomycin solution, 89% dulbecco’s modified eagle medium (DMEM), and 1.0 × 10^4^ human umbilical vein endothelial cells (HUVECs) were added into each sponge and incubated at 37 ℃ incubator with 5% CO_2_. After 1-, 3-, and 5-days’ co-culture, the sponges were washed with phosphate buffer saline (PBS) three times and incubated with the mixture of 40 μL CCK-8 and 360 μL DMEM for 1 h. Then 100 μL suspension was transferred to 96 wells plate and the absorbance was measured at 450 nm to evaluate the cytotoxicity of these sponges. Additionally, after 5 day’s co-culturing, HUVECs on these sponges were fixed with 4% paraformaldehyde for 0.5 h and stained with fluorescein isothiocyanate (FITC)-labeled phalloidin and 4′,6-diamidino-2-phenylindole (DAPI) to observe the cytoskeleton structure and morphology by confocal laser scanning microscope. To further observation the morphology of single cells, after 5 day’s co-culture, HUVECs on these sponges were fixed by the 2.5% glutaraldehyde, followed by the gradient ethanol dehydration (25%, 50%, 75%, 95%, 100%), tert-butanol replacement, and freeze-drying. Finally, the morphology of single cells was observed by the FE-SEM. Besides, the protein expression level of HIF-1α in HUVECs treated with sponges (BNC, OBNC, and OBNC-DFO) was explored by the enzyme-linked immunosorbent assay (ELISA, mlbio, China).
6. **The coagulation experiment in vivo**
  The animal experiment was approved by the Animal Research Committee of Ruijin Hospital, Shanghai Jiaotong University School of Medicine. The male Sprague Dawley (SD) rats (200–250 g) was used in the coagulation and wound healing experiment.
  Two experimental models, the liver trauma and rat tail amputation model, were used to evaluate sponges’ hemostatic effect in vivo. Twenty-four SD rats were randomly divided into four groups (BNC, OBNC, OBNC-DFO, and COL groups). There were 6 rats in each group, 3 of which were used for liver trauma model and 3 were used for tail amputation model. The rats were anesthetized by intraperitoneal injection with 1.0% pentobarbital sodium (40 mg/kg). After the abdominal incision, the liver was exposed. The liver trauma was established by acupuncture with a needle (20 gauge), followed by covered with the pre-weight sponges until blood coagulation. After that, the sponges were weighted again. As for the rat tail amputation model, 50% length of the rat tail was cut, then the incision was covered with pre-weight sponges. After blood coagulation, the sponges were weighted. According to the weight change, the blood loss in these two models was calculated. Meanwhile, the morphologies of sponges before and after absorbing blood were recorded to analyze the percentage of blood-absorbed area.
7. **The wound healing in vivo**
  Twelve male SD rats were randomly divided into four groups and sampled at different time periods (7, 14, 21 and 28 days). Four of full-skin defected models with a diameter of 10 mm were established on each back of rat, treated with BNC, OBNC and OBNC-DFO sponge respectively, and the remaining one wound was served as a control without any treatment. After 0, 7-, 14-, 21-, and 28-days’ treatments, the wound area was recorded, and the following formulation calculated the wound closure rate: wound closure rate (%) = (S0-St)/S0 × 100. S0 indicated the wound closure area on day 0, St revealed the wound closure area on the recorded day. Afterward, the tissues were collected and processed by the paraffin section. The specimens were respectively treated with Hematoxylin and Eosin (HE) staining and Masson staining to evaluate the inflammation and collagen deposition on the wound area. Additionally, immunofluorescence staining, such as CD31, α-SMA, Collagen I, and CK14, was used to investigate the neovascularization, mature blood vessel, the Collagen I deposition, and epithelialization during the wound healing.

### Statistical analysis

All data in the figures were presented as mean ± standard deviation. One-way analysis of variance (ANOVA) was used for statistical analysis to assess the significant differences among the groups. (*) p < 0.01 mean significant difference were detected, and (#) p > 0.05 were considered as no significant difference were detected.

## Supplementary Information

**Additional file 1**. Video of OBNC sponge compression process.

## Data Availability

All data generated or analysed during this study are included in this article.

## Abbreviations

BNC: Bacterial nanocellulose
OBNC: Oxidized bacterial nanocellulose
TEMPO: Tetramethylpiperidine nitroxide
DFO: Deferoxamine
HIF-1α: Hypoxia inducible factor-1α
COL: Collagen
ORC: Oxidized regenerated cellulose
FGF-2: Fibroblast growth factor
VEGF: Vascular endothelial growth factor
PDGFs: Platelet-derived growth factors
EGF: Epidermal growth factor
HUVECs: Human umbilical vein endothelial cells
HE: Hematoxylin and eosin
FTIR: Fourier transform infrared
FE-SEM: Field emission scanning electron microscope
SEM: Scanning electron microscope
PPP: Platelet-poor plasma
H-M time: Half of the maximum time
CCK-8: Cell counting kit-8
FITC: Fluorescein isothiocyanate
DAPI: 4′,6-Diamidino-2-phenylindole
FBS: Fetal bovine serum
DMEM: Dulbecco’s modified eagle medium
ELISA: Enzyme-linked immunosorbent assay
SD rat: Sprague Dawley rat
α-SMA: Alpha smooth muscle actin
